# Improvement of mosquito identification by MALDI-TOF MS biotyping using protein signatures from two body parts

**DOI:** 10.1186/s13071-018-3157-1

**Published:** 2018-11-03

**Authors:** Anubis Vega-Rúa, Nonito Pagès, Albin Fontaine, Christopher Nuccio, Lyza Hery, Daniella Goindin, Joel Gustave, Lionel Almeras

**Affiliations:** 1 Laboratory of Vector Control Research, Environment and Health Unit, Institut Pasteur de la Guadeloupe, 97183 Les Abymes, Guadeloupe France; 2 CIRAD, UMR ASTRE, F-97170 Petit Bourg, Guadeloupe France; 3 ASTRE, CIRAD, INRA, University of Montpellier, Montpellier, France; 4 Unité de Parasitologie et Entomologie, Département des Maladies Infectieuses, Institut de Recherche Biomédicale des Armées, Marseille, France; 5 Aix Marseille Université, IRD, AP-HM, SSA, UMR Vecteurs - Infections Tropicales et Méditerranéennes (VITROME), IHU - Méditerranée Infection, 19–21 bd Jean Moulin, 13385 Marseille, cedex 5, France; 6 Aix Marseille Université, INSERM, SSA, IRBA, MCT, 13005 Marseille, France; 7 Vector Control Service of Guadeloupe, Regional Health Agency, Airport Zone South Raizet, 97139 Les Abymes, Guadeloupe France

**Keywords:** Mosquitoes, Culicidae, Identification, Guadeloupe, MALDI-TOF MS, Innovative strategy

## Abstract

**Background:**

Matrix-assisted laser desorption/ionization time-of-flight mass spectrometry technology (MALDI-TOF MS) is an innovative tool that has been shown to be effective for the identification of numerous arthropod groups including mosquitoes. A critical step in the implementation of MALDI-TOF MS identification is the creation of spectra databases (DB) for the species of interest. Mosquito legs were the body part most frequently used to create identification DB. However, legs are one of the most fragile mosquito compartments, which can put identification at risk. Here, we assessed whether mosquito thoraxes could also be used as a relevant body part for mosquito species identification using a MALDI-TOF MS biotyping strategy; we propose a double DB query strategy to reinforce identification success.

**Methods:**

Thoraxes and legs from 91 mosquito specimens belonging to seven mosquito species collected in six localities from Guadeloupe, and two laboratory strains, *Aedes aegypti* BORA and *Aedes albopictus* Marseille, were dissected and analyzed by MALDI-TOF MS. Molecular identification using *cox*1 gene sequencing was also conducted on representative specimens to confirm their identification.

**Results:**

MS profiles obtained with both thoraxes and legs were highly compartment-specific, species-specific and species-reproducible, allowing high identification scores (log-score values, LSVs) when queried against the in-house MS reference spectra DB (thorax LSVs range: 2.260–2.783, leg LSVs range: 2.132–2.753).

**Conclusions:**

Both thoraxes and legs could be used for a double DB query in order to reinforce the success and accuracy of MALDI-TOF MS identification.

**Electronic supplementary material:**

The online version of this article (10.1186/s13071-018-3157-1) contains supplementary material, which is available to authorized users.

## Background

Despite centuries of control efforts, the past three decades have witnessed a dramatic spread of many mosquito-borne diseases worldwide. Today, they constitute a major public health problem accounting for more than 1.5 million deaths per year [1, 2]. This burden irrefutably demonstrates the need for appropriate mosquito surveillance programmes where specimens are accurately identified at the species level. Mosquito species are primarily identified using morphological traits and dichotomous keys. This identification approach is limited by the damage to the specimens, morphological interspecies similarities, availability of an appropriate identification key and entomological skills [3]. In cases with such limitations, PCR-based methods have proved their efficacy as they can be used with damaged specimens and allow discrimination of morphologically undistinguishable mosquito species and closely related species groups (i.e. *Culex pipiens* form *pipiens*, *Culex pipiens* form *molestus* and hybrids) [3, 4]. However, molecular approaches are expensive and time-consuming, limiting large-scale implementation in the frame of entomological surveillance [5]. In addition, molecular identification requires information on gene target sequences that are frequently unavailable in the corresponding databases. In this context, the use of an alternative cheap and rapid tool, allowing for large scale and high-quality monitoring of culicid populations, is required to revolutionize entomological surveillance.

Matrix-assisted laser desorption/ionization time-of-flight mass spectrometry technology (MALDI-TOF MS) has recently emerged as an innovative tool that has been shown to be effective for rapid and low-cost identification of numerous arthropod groups, including mosquitoes (Culicidae) [6, 7], phlebotomine flies (Psychodidae) [8–10], tsetse flies (Glossinidae) [11], biting midges (Ceratopogonidae) [12], fleas (Siphonaptera) [13] and hard ticks (Ixodidae) [14, 15]. In addition, the sensitivity and specificity of MALDI-TOF MS biotyping allow a correct identification of immature arthropod stages [16, 17] and closely related species, which would otherwise be indistinguishable using morphological and/or molecular approaches (i.e. cryptic *Anopheles gambiae* species) [18].

The efforts conducted over the past five years have yielded improvements to protocols and standardization of methods for this kind of protein-based identification [19, 20]. These guidelines should facilitate the comparison and exchange of MS spectra between teams all over the world. For the mosquito identification at adult stages, legs were repeatedly chosen for the creation of MS reference spectra databases and specimens identification [6, 7, 21]. However, mosquito legs are breakable and the loss of one or several legs occurs frequently during mosquito sampling, transportation or storage. If only three or fewer legs are available for a single specimen, the identification by MALDI-TOF MS could be compromised [22]. In such cases, the selection of another adult mosquito body part for MALDI-TOF MS analysis could solve species identification when failure occurred with mosquito legs.

The abdomen is generally excluded for MS specimen identification due to the high heterogeneity of MS profiles generated by this body part. Indeed, according to the gravid or feeding status (unfed, recently fed or blood meal under digestion) of arthropods, abdomen MS profiles could be drastically different among specimens from the same species [12, 18, 23]. Moreover, MS profiles from freshly engorged mosquitoes can differ according to the source of the recently ingested blood meal [23, 24]; consequently, the mosquito abdomen cannot be considered as a suitable body part for species identification using MALDI-TOF. Because the mosquito head is frequently used to evaluate vector competence (i.e. efficient dissemination of pathogens beyond the midgut barrier) [25–27], the only remaining body part which is not prone to degradation during collection, transportation or storing and that could be used for species identification is the thorax.

The aim of the present study was to assess whether thoraxes from adult mosquitoes can produce species-specific protein signatures that can be used for mosquito species identification, as previously reported for legs (species reproducibility and species specificity of MS spectra). The creation of an MS spectra reference database containing a double entry for queried paired-MS spectra from each specimen is likely to improve user confidence in this method. This may result in popularization of this innovative strategy for entomological studies.

## Results

### Morphological identification and molecular confirmation

**Fig. 1:**
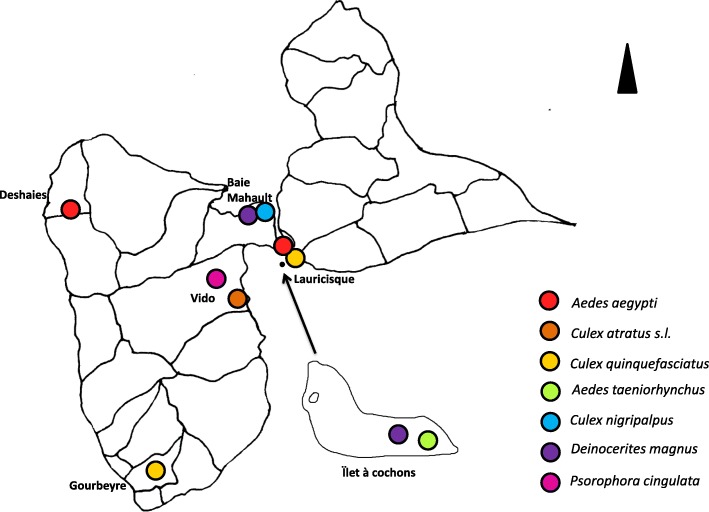
Map of mosquito collection sites and species found per site in Guadeloupe

Among mosquitoes trapped in 6 distinct sites from Guadeloupe (Fig. 1), eight specimens were selected per species and collection site. These mosquitoes were morphologically identified as seven distinct mosquito species (Table 1). Two *Aedes* species, *Ae. aegypti* and *Ae. albopictus*, reared in laboratory were also added as controls. A total of 91 mosquito specimens were included in the present study.

**Table 1:** Overview of mosquito origins and subgroup identification by *cox*1 molecular typing

Morphological identification	Locality	No. of specimens included (sequenced)	Species identified *via* GenBank (accession number)	*cox*1 sequence coverage (%)/similarity (%)	Species identified *via* BOLD	*cox*1 sequence similarity (%)
*Ae. aegypti*	Deshaies, Lauricisque	16 (4)	*Ae. aegypti* (AY432106.1)	92/100	*Ae. aegypti*	100
*Cx. quinquefasciatus*	Gourbeyre, Lauricisque	15^b^ (4)	No reliable ID	–	*Cx. quinquefasciatus*	98.9
*Cx. nigripalpus*	Baie Mahault	8 (2)	*Cx. nigripalpus* (KM592992.1)	87/99	*Cx. nigripalpus*	99.7
*Cx. atratus* (*s.l.*)	Vido	8 (2)	No reliable ID^a^	–	No reliable ID	–
*D. magnus*	Baie Mahault, Ilet à cochons	16 (4)	No reliable ID^a^	–	*D. magnus*	99.4
*Ae. taeniorhynchus*	Ilet à cochons	8 (2)	*Ae. taeniorhynchus* (JX259676.1)	88/99	*Ae. taeniorhynchus*	99.0
*P. cingulata*	Vido	8 (2)	*P. cingulata* (KM592989.1)	88/99	*P. cingulata*	99.1
*Ae. aegypti* (Bora)	Laboratory reared	8 (–)	–	–	–	
*Ae. albopictus* (MRS)	Laboratory reared	4 (–)	–	–	–	

^a^Mosquito species for which *cox*1 sequences were not available in the database (30th March 2018)

^b^Specimens identified morphologically as *Cx. quinquefasciatus* at Lauricisque (*n* = 7) and Gourbeyre (*n* = 8)

*Abbreviations*: No reliable ID, similarity with top match < 97%; BOLD, Barcode of Life Data Systems; *cox*1, cytochrome *c* oxidase subunit 1; MRS, Marseille strain

Two out of eight morphologically identified specimens per species and collection site (25%) were submitted to *cox*1 gene sequencing to confirm their identification. The absence of *cox*1 sequences for *Cx. atratus* and *Deinocerites magnus* on GenBank resulted in unreliable identification (similarity with top match < 97%); *cox*1 sequences obtained for *Cx. atratus* and *D. magnus* were deposited in the GenBank database as new sequences with accession numbers MH376749/MH376750 and MH376751/MH376752, respectively. The query of the remaining *cox*1 sequences in the GenBank database, using the BLAST function, allowed us to obtain reliable mosquito species identification for 10 out of 14 samples, with sequence coverage and identity ranges of 87–92% and 99–100%, respectively (Table 1). The four specimens morphologically identified as *Cx. quinquefasciatus* did not reach the identity sequence threshold of 97% for reliable classification. The first two top-ranking hits of species identification were *Cx. p. pipiens* and *Cx. quinquefasciatus*, both obtaining 96% of *cox*1 sequence similarity. These specimens were therefore classified as *Culex* genus.

In the Barcode of Life Data Systems (BOLD) database, *cox*1 sequences from the seven mosquito species were available (Table 1). The query of the *cox*1 sequences from specimens of six mosquito species corroborated morphological identification, notably for *D. magnus* (similarity > 99.4%) and *Cx. quinquefasciatus* specimens (similarity > 98.9%), and confirmed the four other species identified using the sequences in the GenBank DB. The *cox*1 sequences of specimens morphologically identified as *Cx. atratus* failed to match any species in the BOLD system. The high similarity (> 99% on 649 bp) of *cox*1 sequences from specimens morphologically identified as *Cx. atratus* supported that these mosquito specimens were conspecific. The alignment of *cox*1 sequences of *Cx. atratus* collected in Guadeloupe with those from BOLD revealed a low similarity rate (< 89% on 649 bp). The description of several members in the *Cx. atratus* complex could explain *cox*1 sequence heterogeneity [28]. Based on the lack of specific molecular sequences distinguishing species from *Cx. atratus* complex and the absence of diagnostic morphological character states for female mosquitoes, we classified these specimens as *Cx. atratus* (*s.l.*).

### Reproducible and specific MS spectra from both mosquito body parts

**Fig. 2:**
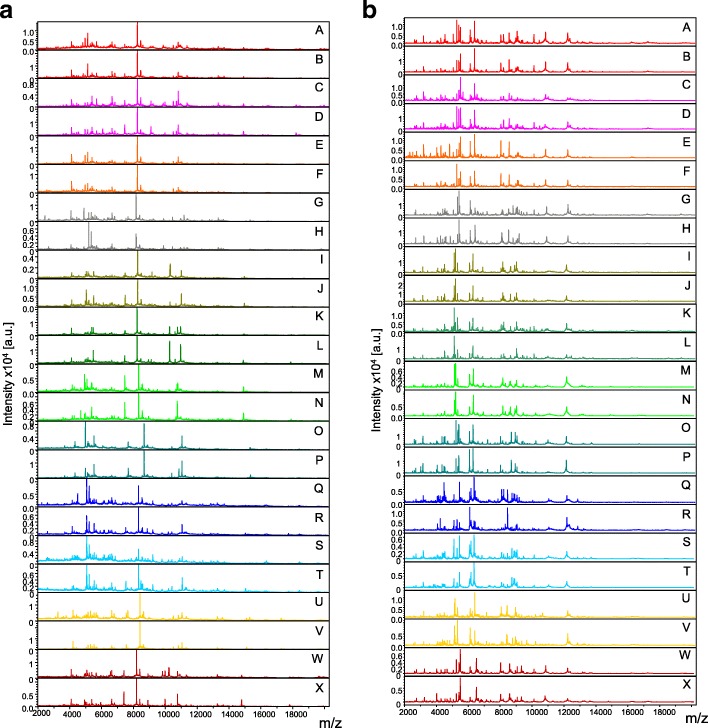
Comparison of MALDI-TOF MS spectra for legs (**a**) and thoraxes (**b**) of mosquitoes. Representative MS spectra of *Ae. aegypti* (Bora) laboratory-reared (A, B) or collected at Deshaies (C, D) and Lauricisque (E, F), *Ae. taeniorhynchus* collected at Ilet à cochons (G, H), *Cx. quinquefaciatus* collected at Gourbeyre (I, J) and Lauricisque (K, L), *Cx. nigripalpus* collected at Baie Mahault (M, N), *Cx. atratus* (*s.l.*) collected at Vido (O, P), *D. magnus* collected at Ilet à cochons (Q, R) and Baie Mahault (S, T), *P. cingulata* collected at Vido (U, V) and *Ae. albopictus* (Marseille strain) laboratory-reared (W, X). *Abbreviations*: a.u., arbitrary units; m/z, mass to charge ratio

Legs and thoraxes from each of the 91 mosquitoes were dissected prior to MS analysis. Unfortunately, legs from 3 specimens (1 *Cx. atratus* and 2 *Cx. quinquefasciatus*) were missing, probably broken during transport and/or storing, and one thorax from *Psorophora cingulata* was lost during the dissection step. Finally, legs and thoraxes from 88 and 90 specimens, respectively, were submitted to MALDI-TOF MS analysis. The comparison of MS spectra from legs (Fig. 2a) and thoraxes (Fig. 2b) between mosquito species revealed protein profiles of high intensity (> 2000 a.u.) and were visually reproducible for specimens of the same species according to body part.

**Fig. 3:**
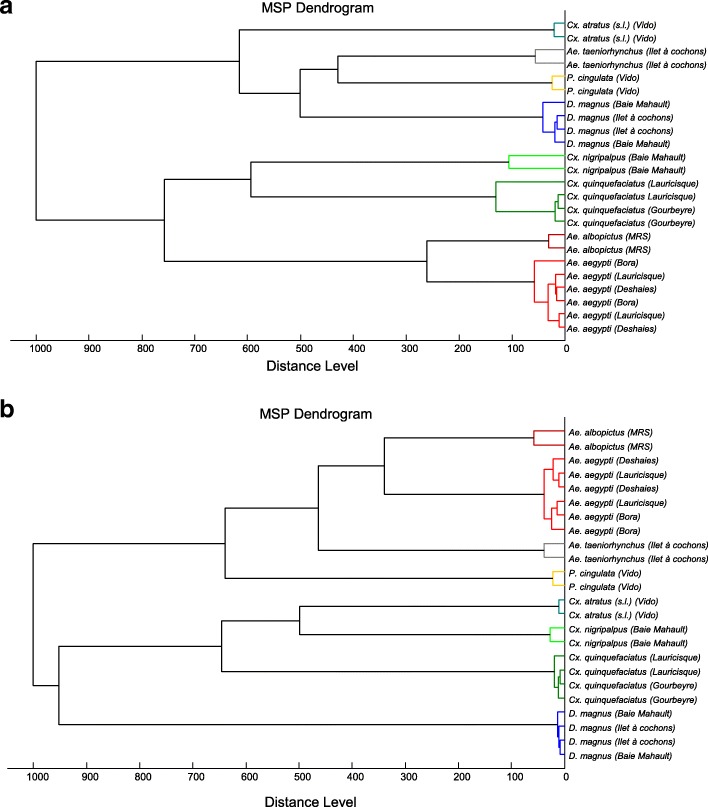
MSP dendrogram of MALDI-TOF MS spectra for legs (**a**) and thoraxes (**b**) of mosquitoes. Two specimens per collection site were used to construct the dendrogram. The dendrogram was created using Biotyper v3.0 software and distance units correspond to the relative similarity of MS spectra. Mosquito sampling locations are given within parentheses. *Abbreviation*: MRS, Marseille

To assess the reproducibility and specificity of the MS spectra from legs and thoraxes per species according to body parts, a cluster analysis was performed. Two specimens per species and site plus both *Aedes* laboratory-reared species were used for building MSP dendrograms. The clustering of leg (Fig. 3a) and thorax (Fig. 3b) MS spectra according to mosquito species confirmed the reproducibility and specificity of the protein profiles. The clustering of specimens of the same species, independent of the trapping location site or origin (field or laboratory reared), in each body part, confirmed the high species-specificity of MS spectra. Furthermore, a total of 54 and 56 species-specific mass peaks were found with ClinProTools software between these mosquito species for legs and thoraxes, respectively (Additional file 1: Table S1 and Additional file 2: Table S2). MS spectra generated from legs, thoraxes and a mix of legs and thoraxes from the laboratory reared mosquito species *Ae. albopictus* were also compared (Additional file 3: Figure S1a, b). Interestingly, MS spectra from mixed compartments (i.e. legs and thorax) were similar to thorax counterparts. The more abundant *Ae. albopictus* MS peak obtained with legs (m/z = 8191.5) was nearly undetectable in the MS profiles from mixed legs and thoraxes (Additional file 3: Figure S1c, d).

It is interesting to note that MSP dendrogram generated with mosquito paired body parts were not superimposable between legs and thoraxes (Fig. 3). All specimens of the genus *Culex* were grouped in the same part of the MSP dendrogram for thoraxes, whereas for the leg MSP dendrogram, *Cx. atratus* specimens were isolated from other mosquitoes of the genus. Moreover, *Ae. taeniorhynchus*, a species from the genus *Aedes*, was found at close proximity to other members of this genus solely in the MSP dendrogram from thoraxes.

**Fig. 4:**
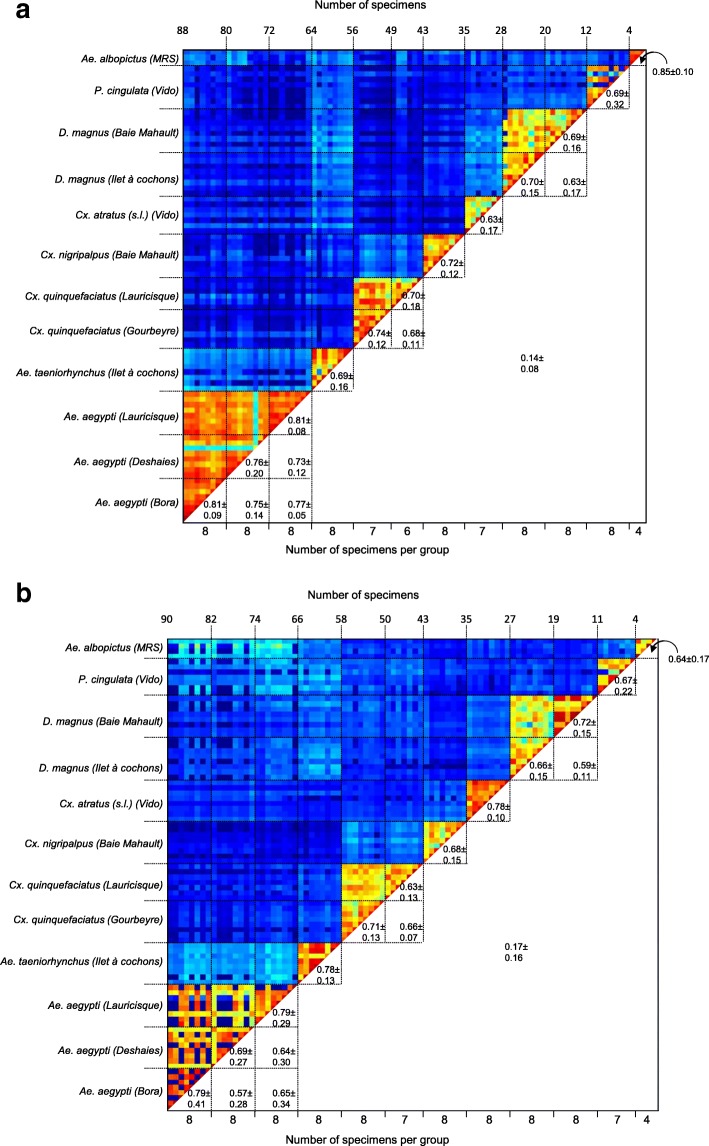
Assessment of the reproducibility of MS spectra for legs (**a**) and thoraxes (**b**) according to mosquito species using the composite correlation index (CCI). MS spectra from four to eight specimens per collection site were analysed using the CCI tool. Levels of MS spectra reproducibility are indicated in red and blue showing relatedness and incongruence between spectra, respectively. CCI matrix was calculated using MALDI-Biotyper v3.0 software with default settings (mass range 3.0–12.0 kDa; resolution 4; 8 intervals; auto-correction off). The values correspond to the mean coefficient correlation and respective standard deviations obtained for paired condition comparisons. CCI are expressed as the mean ± standard deviation. Mosquito sampling locations are given within parentheses. *Abbreviation*: MRS, Marseille

MS spectra reproducibility from legs and thoraxes were confirmed by CCI matrix highlighting a good correlation between spectra for specimens of the same species whatever their origin (catching location, field-caught or laboratory-reared). Moreover, the low CCI obtained for the comparisons of MS spectra between species using leg (mean ± SD: 0.14 ± 0.08; Fig. 4a) and thorax (0.17 ± 0.16; Fig. 4b) MS spectra supported the species-specificity of the protein profiles.

Interestingly, paired comparisons of legs and thoraxes MS profiles for each species showed clearly distinct protein patterns (Fig. 2). To visualize specificity of the MS spectra according to body part per species, principal components analyses (PCAs) were performed (Additional file 4: Figure S2). PCAs revealed a clear separation of the points corresponding to MS spectra from the legs and thoraxes, confirming a specificity of MS profiles between these two compartments for the seven species tested.

### MS reference spectra database creation and validation step

**Fig. 5:**
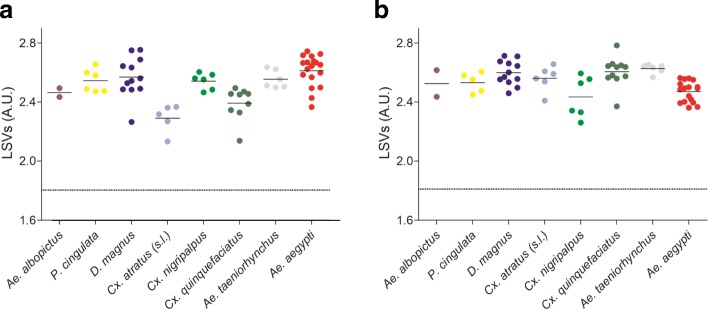
Comparison of LSVs from MS spectra for legs (**a**) and thoraxes (**b**) from all mosquito species studied. Dashed lines represent the threshold value for relevant identification (LSVs > 1.8). *Abbreviation*: LSV, log-score value

MS spectra for legs and thoraxes from the 24 specimens used for MSP cluster analysis, including at least 2 specimens per species and location site, identified morphologically and molecularly, were used as reference MS spectra for the database (DB) creation. The remaining 64 and 66 MS spectra from legs and thoraxes, respectively, were queried against this DB. Log-score values (LSVs) from legs ranged between 2.132–2.753 and from thoraxes ranged between 2.260–2.783. MS spectra were higher than the threshold value (LSVs > 1.8) for reliable identification [6, 20] for all the samples tested (Fig. 5). For paired samples, concordant species identification (100%) was obtained using MS spectra from legs and thoraxes which were in agreement with morphological classification. The four specimens for which only one body part (legs or thoraxes) was submitted to MS, also provided concordant results with entomological identification.

To verify that reliable identification was achieved for the reference MS spectra whatever the collection site of specimens, a comparison of LSVs resulting from a DB query containing MS spectra from two specimens per species and location site or two specimens per species from a single location site, was done. As expected, a decrease of LSVs was observed when only specimens from one site were included in the DB, compared to all sites (Additional file 5: Figure S3). Nevertheless, identification scores remained sufficiently high (LSVs > 1.8) to unambiguously classify all specimens, using either mosquito legs or thoraxes. Interestingly, reliable identification could be made when unique MS spectra from legs or thoraxes of laboratory-reared *Ae. aegypti* (Bora) were included in the DB (Additional file 5: Figure S3a, b). These results underline that these two body parts can be used for identification, independent of specimen origin, within mosquitoes from the same species.

## Discussion

The present study constitutes a representative example of the requirement for an innovative method for arthropod identification. Although morphological analysis remains the “gold standard” for mosquito identification [22], this time-consuming method is reliant on entomologist skills and mosquito integrity which are drawbacks for its widespread use. In regions such as the Caribbean, morphological identification is even more complex due to a generalized lack of recent culicid fauna inventories (most of them were conducted in the 1960s and 1970s) [29, 30] and updated identification keys. Effectively, the absence of morphological dichotomous keys adapted to local fauna impedes the correct morphological diagnosis of closely-related species such as members belonging to a species complex. Moreover, for these closely-related species (i.e. *Culex* spp.), dissection steps (i.e. male genitalia) are often required for a reliable identification at species level, due to the absence of discriminative external morphological characters [31]. Well-trained personnel possessing expertise is, therefore, critical for successful morphological identification. In the present study, initial morphological observation failed to confidently categorize female specimens from the *Cx. atratus* complex, which were then classified as *Cx. atratus* (*s.l.*). In the USA, the distinction of *Cx. atratus* (*s.s.*) from *Cx. atratus* B is based on the inspection of the cibarial armature, a small structure located at the base of the pharyngeal pump, requiring mosquito head dissection [28]. Two types of cibarial armatures were described for each member of *Cx. atratus* complex, increasing the risk of misidentification [28]. In addition, species composition of the *Cx. atratus* complex in the Neartic region (e.g. the USA) is different to that of the Neotropical region (e.g. the Caribbean). Therefore, it was uncertain at the time the study was implemented to discern whether different cibarial armature states could be confidently associated with a particular species of the Neotropical *Cx. atratus* complex, as other local species from the complex were unavailable.

To circumvent the limitations of morphological identification, molecular strategies based on DNA-barcoding using *cox*1 gene sequencing are generally applied [32, 33]. However, this expensive and time-consuming approach was inefficient in the identification of all specimens collected in Guadeloupe in the frame of the present study. Here, the query of the *cox*1 sequences against the GenBank DB did not successfully identify *Cx. quinquefasciatus* specimens, despite the presence of counterpart *cox*1 sequences. The low percentage of *cox*1 sequences similarity (about 96%) between *Cx. quinquefasciatus* field-collected specimens from Guadeloupe and counterpart species on GenBank allowed identification solely to the genus level (*Culex* sp.). Conversely, the same *cox*1 sequences queried against the BOLD DB confirmed that these specimens belonged to *Cx. quinquefasciatus* with a relevant similarity rate (98.9%). These results underlined that identification success could be dependent of the genomic DB used. The inability to identify certain species was attribute to the absence of the concerned *cox*1 sequences in the DB [i.e. *Cx. atratus* (*s.l.*)] and to the representativeness of *cox*1 gene diversity within a given species (i.e. *Cx. quinquefasciatus*). Indeed, genetic variations in the *cox*1 gene have been reported between specimens from the same mosquito species (i.e. *Cx. bidens*, *Cx. interfor*) collected in different provinces of Argentina, allowing the identification of different haplotypes [31]. Even if this genetic diversity is lower when compared to that of other genes (i.e. the *nad*4 gene), it can influence the molecular identity rate obtained after DB query as observed here.

MS spectra variations between specimens from the same species with a different geographical origin was also observed. This phenomenon was reported for mosquitoes at the adult [6, 18] and larval stages [16], as well as for other arthropod groups such as tsetse flies [11]. MS profile variations according to mosquito origin were also observed in the present study. It was highlighted by the decrease of LSVs depending on the geographical location of the specimens included in the reference MS DB. Nevertheless, these intraspecific variations of MS spectra were moderate and did not negatively affect the correctness and reliability of counterpart specimen identification. Moreover, the exact identification of field-caught *Ae. aegypti* by inclusion in the reference MS DB of MS spectra from laboratory-reared *Ae. aegypti* (Bora) specimens underlined the robustness of this approach.

For *Cx. atratus* (*s.l.*) and *D. magnus*, *cox*1 sequences were not available in the GenBank DB, which resulted in failure to confirm the morphological identification. The lack of complete *cox*1 sequences in a public genomic DB frequently occurs, requiring the sequencing of other reference genes (i.e. ribosomal), or of several *cox*1 gene fragments [31], when possible, to achieve identification. Conversely, the same *cox*1 sequences queried against the BOLD DB allowed the identification of six out of seven species, confirming the morphological identification, including *D. magnus* specimens. Only specimens morphologically identified as *Cx. atratus* (*s.l.*) failed to be validated with *cox*1 sequence similarity lower than 97% following a query in BOLD. Nevertheless, as observed for *Cx. quinquefasciatus* in the GenBank DB, *Cx. atratus* (*s.l.*) *cox*1 sequences were available in the BOLD DB. However, it was not indicated to which member of the *Cx. atratus* complex corresponded the available *cox*1 sequences, i.e. *Cx. atratus* (*s.s.*) or *Cx. atratus* B [28]. The low *cox*1 sequence similarity (< 89%) between *Cx. atratus* (*s.l.*) specimens from Guadeloupe and those available in the BOLD DB could suggest that the former belong to a different species of the *Cx. atratus* complex. The absence of another gene sequence target allowing to distinguish members of the *Cx. atratus* complex in the GenBank DB or the BOLD DB, did not allow us to definitively identify the specimens collected in Guadeloupe. However, the high homology of the *cox*1 sequences between specimens allowed us to consider them conspecific but as *Cx. atratus* (*s.l.*). The reproducibility of MS spectra, for each compartment, from specimens identified as *Cx. atratus* (*s.l.*), in addition to the elevated CCI obtained for both legs and thoraxes, were supplementary arguments to claim that these specimens could be considered as same species. The high efficiency of MALDI-TOF MS biotyping to distinct cryptic species has been repeatedly reported, notably distinguishing *An. gambiae* (*s.s.*) from *An. coluzzi*, corresponding to *An. gambiae M* and *S* molecular forms, respectively [18, 20, 34]. In the future, this proteomic approach could be evaluated to differentiate members from the *Cx. atratus* complex.

A critical step in the implementation of MALDI-TOF MS identification is the creation of MS reference spectra DB for the species of interest. The majority of the existing adult mosquito identification MS DB have been created using mosquito legs [6]. However, legs are one of the most fragile mosquito compartments and they are easily lost during specimen handling and/or storage, which compromises their identification. In the present study, three specimens had all the legs missing, which required identification based on thorax MS spectra to corroborate morphological classification. Moreover, for several other specimens, one or more legs were missing which could have prejudiced their identification. The use of another mosquito compartment to create MS reference DB as a complement of MS spectra obtained from mosquito legs could be an alternative to counteract this technical problem and reinforce the quality of the identification (Additional file 6: Figure S4). The present study demonstrated that mosquito thoraxes could also be used as a suitable and relevant body part for mosquito species identification using MALDI-TOF MS biotyping strategy.

The comparison of MS profiles obtained with legs, thoraxes and a mix of legs and thoraxes from *Ae. albopictus* specimens revealed similar MS profiles between mixed compartments and thorax counterparts. In MS profiling, mainly abundant ionizable proteins are detectable. As proteins from legs were proportionally less abundant than those from the thorax, their protein quantities were insufficient to drastically change thorax MS profiles. Nevertheless, under the hypothesis that thorax MS profiles would be changed by the addition of legs for other species, it is probable that intensity of MS peaks corresponding to legs in the mixture could be variable according to the number of legs included. In such conditions, the reference MS spectra database from mixed legs and thoraxes would not be suitable to identify specimens which had lost several or all legs. Conversely, when legs were submitted alone, the MS profile was unchanged whatever the number of legs and only a decrease of the MS profile intensity was observed according to the number of legs used. For specimens with one or two legs remaining, the low ratio intensity of MS peaks/background can hinder peak detection, compromising their identification. The MS submission of a second compartment, such as the thorax, could then reveal specimen identity, especially for specimens which have lost several legs or for closely-related species.

This new strategy consisting of submitting two body compartments per specimen to MALDI-TOF MS analysis, thereby producing distinct but species-specific MS spectra, should improve identification confidence. Moreover, the distinct topologies of the MSP dendrograms for each body part from paired specimens, pointed out that the proximity of MS spectra between mosquito species were different for legs and thoraxes. These MS pattern properties reinforce the species identification accuracy and reduce the risk of misidentification. The MS spectra specificity according to body part used, was also reported in others arthropods such as ticks [35] and it has been recommended to submit legs and half-idiosome from the same tick specimen to MS for MS reference DB creation or to strengthen specimen identification [35].

In addition to the standardization of sample preparation protocols [19, 20, 36], the creation of a standardized MS reference spectra DB for mosquito identification composed of paired legs and thoraxes MS spectra for each species becomes compulsory. The double MS spectra query strategy remains compatible with the use of remaining body parts of the specimen to obtain additional information. For instance, the remaining abdomen could still be used to detect the blood meal source in the case of an engorged mosquito female [23], while the head can be used for pathogen detection [36], both by a MALDI-TOF biotyping approach.

## Conclusions

The present study points out MALDI-TOF biotyping strategy as an innovative and alternative tool for mosquito identification that could lead to a dramatic positive impact in the mosquito surveillance field worldwide. The double MS spectra DB query should reinforce identification quality and, in the case of missing legs, identification remains possible by the use of the thorax. To achieve this, the development of an international comprehensive and accessible mosquito spectra database is essential.

## Methods

### Mosquitoes

Adult female mosquitoes, collected in the field or laboratory-reared, were used for this study. Field collection of mosquitoes was undertaken in 6 distinct sites from the Caribbean Island of Guadeloupe using BG sentinel traps (Fig. 1). Collected specimens were stored at -20 °C from a few months to one year. Mosquito species were determined by morphological identification under a binocular loupe at a magnification of 56× (Leica M80, Leica, Nanterre, France) using morphological descriptions [37–39]. In each collection site, 8 specimens per species were selected for MS analysis. *Aedes aegypti* (Bora strain) and *Ae. albopictus* (Marseille strain, MRS) mosquitoes were raised in the laboratory using standard methods as previously described [40, 41]. Only imago non-engorged female mosquitoes were included in the study. The mosquitoes were sedated and stored at -20 °C until future analysis.

### Mosquito dissection

Legs and thoraxes from mosquitoes were processed as previously described [36]. Briefly, specimens were individually dissected with a sterile surgical blade under a binocular loupe. For each specimen, legs and thorax (without wings) were removed and transferred separately in 1.5 ml Eppendorf tubes for MALDI-TOF MS analysis. The remaining body parts (abdomens and heads) were used for molecular analyses. A nomenclature was established to pair body parts from the same specimen. In addition, paired legs and thorax (without wings) from five *Ae. albopictus* specimens were mixed prior to MALDI-TOF MS analysis.

### Molecular identification of mosquitoes

DNA was individually extracted from the head and abdomen of 2 mosquito specimens per species selected for MS reference database creation (*n* = 24) using the QIAamp DNA tissue extraction kit (Qiagen, Hilden, Germany) according to the manufacturer’s instructions. Molecular identification of mosquito at the species level was performed by sequencing the PCR product of a fragment of the cytochrome *c* oxidase 1 gene (*cox*1) using the primers LCO1490 (forward) (5'-GGT CAA CAA ATC ATA AAG ATA TTG G-3') and HC02198 (reverse) (5'-TAA ACT TCA GGG TGA CCA AAA AAT CA-3') as previously described [42, 43]. The sequences were assembled and analyzed using the ChromasPro software version 1.7.7 (Technelysium Pty. Ltd., Tewantin, Australia). All sequences were compared with sequences in the GenBank database using BLAST (http://blast.ncbi.nlm.nih.gov/Blast.cgi) and the Barcode of Life Data Systems (BOLD; http://www.barcodinglife.org; [44]) to assign unknown *cox*1 sequences to mosquito species.

### Sample homogenization and MALDI-TOF MS analysis

Each compartment dissected was individually homogenized 3 × 1 min at 30 Hz using TissueLyser (Qiagen) and glass powder (Sigma-Aldrich, Lyon, France) in a homogenization buffer composed of a mix (50/50) of 70% (v/v) formic acid (Sigma-Aldrich, Lyon, France) and 50% (v/v) acetonitrile (Fluka, Buchs, Switzerland) for protein extraction according to the standardized automated setting described by Nebbak et al. [20]. Respectively, 30 μl and 50 μl of the homogenization buffer were used for legs and thoraxes. For samples containing a mix of legs and thorax from the same individual, 60 μl of the homogenization buffer were used. After sample homogenization, a quick spin centrifugation at 200× *g* for 1 min was then performed and 1 μl of the supernatant of each sample was loaded on the MALDI-TOF steel target plate in quadruplicate (Bruker Daltonics, Wissembourg, France). After air-drying, 1 μl of matrix solution composed of saturated α-cyano-4-hydroxycinnamic acid (Sigma-Aldrich, Lyon, France), 50% (v/v) acetonitrile, 2.5% (v/v) trifluoroacetic acid (Sigma-Aldrich, Dorset, UK) and HPLC-grade water was added. To control matrix quality (i.e. absence of MS peaks due to matrix buffer impurities) and MALDI-TOF apparatus performance, matrix solution was loaded in duplicate onto each MALDI-TOF plate alone and with a bacterial test standard (Bruker Bacterial Test Standard, ref: #8255343). Moreover, legs or thoraxes from two *Ae. albopictus* specimens reared at the laboratory and stored at -20 °C were included on each plate and were used as homogenization positive controls.

### MALDI-TOF MS parameters

Protein mass profiles were obtained using a Microflex LT MALDI-TOF Mass Spectrometer (Bruker Daltonics, Bremen, Germany), with detection in the linear positive-ion mode at a laser frequency of 50 Hz within a mass range of 2–20 kDa. The setting parameters of the MALDI-TOF MS apparatus were identical to those previously used [19]. The spectrum profiles obtained were visualized with Flex analysis v.3.3 software and exported to ClinProTools version v.2.2 and MALDI-Biotyper v.3.0 (Bruker Daltonics, Germany) for data processing (smoothing, baseline subtraction, peak picking) and evaluation with cluster analysis.

### MS spectra analysis

MS spectra profiles were first controlled visually with flexAnalysis v.3.3 software (Bruker Daltonics, Bremen, Germany). MS spectra were then exported to ClinProTools v.2.2 and MALDI-Biotyper v.3.0. (Bruker Daltonics, Bremen, Germany) for data processing (smoothing, baseline subtraction, peak picking). MS spectra reproducibility was assessed by the comparison of the average spectral profiles (main spectrum profile, MSP) obtained from the four spots for each specimen according to body part with MALDI-Biotyper v.3.0 software (Bruker Daltonics, Bremen, Germany). MS spectra reproducibility and specificity taking into account mosquito body part were demonstrated using clustering analyses and the composite correlation index (CCI) tool. In addition, ClinProTools software was used to identify discriminatory peaks among the 8 mosquito species for each body-part. Cluster analyses (MS dendrogram) were performed based on comparison of the MSP given by MALDI-Biotyper v.3.0. software and clustered according to protein mass profile (i.e. their mass signals and intensities). The CCI tool from MALDI-Biotyper v.3.0 software was also used to assess the spectral variations within and between each sample group, according to the body part, as previously described [20, 45]. Higher correlation values (expressed as the mean ± standard deviation, SD) reflecting higher reproducibility for the MS spectra, were used to estimate MS spectra distance between species for each body part. To visualize MS spectra distribution from mosquitoes according to body part, principal components analysis (PCA) from ClinProTools v.2.2 software was performed for each species.

### Database creation and blind tests

The reference MS spectra were created using spectra from legs and thoraxes of two specimens per species collected in each site or reared at the laboratory using MALDI-Biotyper software v.3.0. (Bruker Daltonics, Bremen, Germany) [10]. MS spectra were created with an unbiased algorithm using information on the peak position, intensity and frequency. Raw MS spectra from legs and thoraxes of mosquitoes included in the MS reference database used in the present study are provided for free use (Additional file 7). MS spectra from mosquito legs and thoraxes were tested against the in-house MS reference spectra DB. The reliability of species identification was estimated using the log-score values (LSVs) obtained from the MALDI Biotyper software v.3.0, which ranged from 0 to 3. According to previous studies [6, 20], LSVs greater than 1.8 were considered reliable for species identification. Data were analyzed by using GraphPad Prism software v.5.01 (GraphPad, San Diego, CA, USA).

## Additional files

Additional file 1: **Table S1.** Mass peak list distinguishing mosquito species using legs as biological material, based on the Genetic Algorithm model analysis of ClinProTools. The list includes unique species-specific mass peaks. *Abbreviations*: Da, Daltons; m/z, mass to charge ratio. (DOCX 16 kb)

Additional file 2: **Table S2.** Mass peak list distinguishing mosquito species using thoraxes as biologic material, based on the Genetic Algorithm model analysis of ClinProTools. The list includes unique species-specific mass peaks. *Abbreviations*: Da, Daltons; m/z, mass to charge ratio. (DOCX 17 kb)

Additional file 3: **Figure S1.** Resulting MS spectra for legs (red), thoraxes (green) and mix of legs and thoraxes (blue) from *Ae. albopictus* specimens. Five specimens per condition, loaded in quadruplicate, were tested. **a** Overlay of the mean MS profile per condition. **b** Gel view of the MS profiles per condition. An enlargement of the m/z window including the more intense MS peak from *Ae. albopictus* legs is presented as a MS spectra overlay (**c**), and gel view (**d**). (TIF 23971 kb)

Additional file 4: **Figure S2.** Principal components analysis (PCA) from MS spectra for mosquito legs and thoraxes. PCA 3-dimensional (PCA1-PCA3) image from MS spectra of legs (red dots) and thoraxes (green dots) from *Ae. aegypti* (**a**), *Cx. quinquefasciatus* (**b**), *Ae. taeniorynchus* (**c**), *P. cingulata* (**d**), *Cx. atratus* (*s.l.*) (**e**), *Cx. nigripalpus* (**f**) and *D. magnus* (**g**). (TIF 44631 kb)

Additional file 5: **Figure S3.** Geographical reproducibility of the MS spectra from legs (**a**, **c**, **e**) and thoraxes (**b**, **d**, **f**) included in the DB per mosquito species. LSVs obtained for *Ae. aegypti* (**a**, **b**), *Cx. quinquefasciatus* (**c**, **d**) and *D. magnus* (**e**, **f**), according to origins of the MS spectra from specimens of the same species included in the DB are shown. The dashed line represents the threshold value for relevant identification (LSVs > 1.8). *Abbreviation*: LSV, log-score value. (TIF 35156 kb)

Additional file 6: **Figure S4.** Experimental design for mosquito identification using two distinct compartments by MALDI-TOF MS. The advantages of the creation of a MS reference database (DB) including mosquito legs and thoraxes are indicated. (PDF 95 kb)

Additional file 7: Raw MS spectra from legs and thoraxes of mosquitoes included in the MS reference database. MS spectra were obtained using a Microflex LT MALDI-TOF Mass Spectrometer (Bruker Daltonics). (7Z 4576 kb)

## Abbreviations

CCI: Composite correlation index
LSV: Log-score value
MALDI-TOF MS: Matrix-assisted laser desorption/ionization time-of-flight mass spectrometry
PCR: Polymerase chain reaction

## References

[1] WHO (2014). Global Health Day: about vector borne diseases.

[2] UNICEF/WHO. Reversing the incidence of malaria 2000–2015. WHO Global Malaria Program. Geneva: World Health Organization. p. 2015. http://apps.who.int/iris/bitstream/10665/184521/1/9789241509442_eng.pdf?ua=1. Accessed 21 May 2018

[3] Kent RJ, Deus S, Williams M, Savage HM (2010). Development of a multiplexed polymerase chain reaction-restriction fragment length polymorphism (PCR-RFLP) assay to identify common members of the subgenera *Culex* (*Culex*) and *Culex* (*Phenacomyia*) in Guatemala. Am J Trop Med Hyg..

[4] Amraoui F, Tijane M, Sarih M, Failloux A-B (2012). Molecular evidence of *Culex pipiens* form *molestus* and hybrids *pipiens*/*molestus* in Morocco, North Africa. Parasit Vectors.

[5] Freiwald A, Sauer S (2009). Phylogenetic classification and identification of bacteria by mass spectrometry. Nat Protoc.

[6] Yssouf A, Parola P, Lindström A, Lilja T, L’Ambert G, Bondesson U (2014). Identification of European mosquito species by MALDI-TOF MS. Parasitol Res.

[7] RAHARIMALALA F. N., ANDRIANINARIVOMANANA T. M., RAKOTONDRASOA A., COLLARD J. M., BOYER S. (2017). Usefulness and accuracy of MALDI-TOF mass spectrometry as a supplementary tool to identify mosquito vector species and to invest in development of international database. Medical and Veterinary Entomology.

[8] Dvorak V, Halada P, Hlavackova K, Dokianakis E, Antoniou M, Volf P (2014). Identification of phlebotomine sand flies (Diptera: Psychodidae) by matrix-assisted laser desorption/ionization time of flight mass spectrometry. Parasit Vectors.

[9] Mathis A, Depaquit J, Dvořák V, Tuten H, Bañuls A-L, Halada P (2015). Identification of phlebotomine sand flies using one MALDI-TOF MS reference database and two mass spectrometer systems. Parasit Vectors..

[10] Lafri I, Almeras L, Bitam I, Caputo A, Yssouf A, Forestier CL (2016). Identification of Algerian field-caught phlebotomine sand fly vectors by MALDI-TOF MS. PLoS Negl Trop Dis.

[11] Hoppenheit A, Murugaiyan J, Bauer B, Steuber S, Clausen PH, Roesler U (2013). Identification of tsetse (*Glossina* spp.) using matrix-assisted laser desorption/ionisation time of flight mass spectrometry. PLoS Negl Trop Dis.

[12] Kaufmann C, Schaffner F, Ziegler D, Pflüger V, Mathis A (2012). Identification of field-caught *Culicoides* biting midges using matrix-assisted laser desorption/ionization time of flight mass spectrometry. Parasitology.

[13] Yssouf A, Socolovschi C, Leulmi H, Kernif T, Bitam I, Audoly G (2014). Identification of flea species using MALDI-TOF/MS. Comp. Immunol Microbiol Infect Dis.

[14] Karger A, Kampen H, Bettin B, Dautel H, Ziller M, Hoffmann B (2012). Species determination and characterization of developmental stages of ticks by whole-animal matrix-assisted laser desorption/ionization mass spectrometry. Ticks Tick Borne Dis.

[15] Kumsa B, Laroche M, Almeras L, Mediannikov O, Raoult D, Parola P (2016). Morphological, molecular and MALDI-TOF mass spectrometry identification of ixodid tick species collected in Oromia, Ethiopia. Parasitol Res.

[16] Dieme C, Yssouf A, Vega-Rúa A, Berenger J-M, Failloux A-B, Raoult D (2014). Accurate identification of Culicidae at aquatic developmental stages by MALDI-TOF MS profiling. Parasit Vectors.

[17] Suter T, Flacio E, Fariña BF, Engeler L, Tonolla M, Müller P (2015). First report of the invasive mosquito species *Aedes koreicus* in the Swiss-Italian border region. Parasit Vectors.

[18] Müller P, Pflüger V, Wittwer M, Ziegler D, Chandre F, Simard F (2013). Identification of cryptic *Anopheles* mosquito species by molecular protein profiling. PLoS One.

[19] Nebbak A, El Hamzaoui B, Berenger JM, Bitam I, Raoult D, Almeras L (2017). Comparative analysis of storage conditions and homogenization methods for tick and flea species for identification by MALDI-TOF MS. Med Vet Entomol.

[20] Nebbak A, Willcox AC, Bitam I, Raoult D, Parola P, Almeras L (2016). Standardization of sample homogenization for mosquito identification using an innovative proteomic tool based on protein profiling. Proteomics.

[21] Yssouf Amina, Socolovschi Cristina, Flaudrops Christophe, Ndiath Mamadou Ousmane, Sougoufara Seynabou, Dehecq Jean-Sebastien, Lacour Guillaume, Berenger Jean-Michel, Sokhna Cheikh Sadibou, Raoult Didier, Parola Philippe (2013). Matrix-Assisted Laser Desorption Ionization - Time of Flight Mass Spectrometry: An Emerging Tool for the Rapid Identification of Mosquito Vectors. PLoS ONE.

[22] Yssouf A, Almeras L, Raoult D, Parola P (2016). Emerging tools for identification of arthropod vectors. Future Microbiol..

[23] Niare S, Berenger J-M, Dieme C, Doumbo O, Raoult D, Parola P (2016). Identification of blood meal sources in the main African malaria mosquito vector by MALDI-TOF MS. Malar J.

[24] Niare Sirama, Tandina Fatalmoudou, Davoust Bernard, Doumbo Ogobara, Raoult Didier, Parola Philippe, Almeras Lionel (2018). Accurate identification of Anopheles gambiae Giles trophic preferences by MALDI-TOF MS. Infection, Genetics and Evolution.

[25] Fansiri T, Fontaine A, Diancourt L, Caro V, Thaisomboonsuk B, Richardson JH (2013). Genetic mapping of specific interactions between *Aedes aegypti* mosquitoes and dengue viruses. PLoS Genet.

[26] Vega-Rua A, Zouache K, Girod R, Failloux AB, Lourenco-de-Oliveira R (2014). High level of vector competence of *Aedes aegypti* and *Aedes albopictus* from ten American countries as a crucial factor in the spread of chikungunya virus. J Virol.

[27] Chouin-Carneiro T, Vega-Rua A, Vazeille M, Yebakima A, Girod R, Goindin D (2016). Differential susceptibilities of *Aedes aegypti* and *Aedes albopictus* from the Americas to Zika virus. PLoS Negl Trop Dis.

[28] Williams MR, Savage HM (2009). Identification of *Culex* (*Melanoconion*) species of the United States using female cibarial armature (Diptera: Culicidae). J Med Entomol..

[29] Belkin JN, Heinemann SJ (1975). Collection records of the project “Mosquitoes of Middle America.” 3. Bahama Is. (BAH), Cayman Is. (CAY), Cuba (CUB), Haiti (HAC, HAR, HAT) and Lesser Antilles (LAR). Mosq Syst.

[30] Belkin JN, Heinemann SJ (1976). Collection records of the project “Mosquitoes of Middle America.” 4. Leeward Islands: Anguilla (ANG), Antigua (ANT), Barbuda (BAB), Montserrat (MNT), Nevis (NVS), St. Kitts (KIT). Mosq Syst.

[31] Laurito M, Ayala AM, Almirón WR, Gardenal CN (2017). Molecular identification of two *Culex* (*Culex*) species of the neotropical region (Diptera: Culicidae). PLoS One.

[32] Versteirt V, Nagy ZT, Roelants P, Denis L, Breman FC, Damiens D (2015). Identification of Belgian mosquito species (Diptera: Culicidae) by DNA barcoding. Mol Ecol Resour.

[33] Batovska J, Blacket MJ, Brown K, Lynch SE (2016). Molecular identification of mosquitoes (Diptera: Culicidae) in southeastern Australia. Ecol Evol.

[34] Sawadago S, Costantini C, Pennetier C, Diabate A, Gibson G, Dabire R (2013). Differences in timing of mating swarms in sympatric populations of *Anopheles coluzzii* and *Anopheles gambiae s.s.* (formerly *An. gambiae* M and S molecular forms) in Burkina Faso, West Africa. Parasit Vectors.

[35] Boyer Pierre H., Boulanger Nathalie, Nebbak Amira, Collin Elodie, Jaulhac Benoit, Almeras Lionel (2017). Assessment of MALDI-TOF MS biotyping for Borrelia burgdorferi sl detection in Ixodes ricinus. PLOS ONE.

[36] Tahir D, Almeras L, Varloud M, Raoult D, Davoust B, Parola P (2017). Assessment of MALDI-TOF mass spectrometry for filariae detection in *Aedes aegypti* mosquitoes. PLoS Negl Trop Dis..

[37] Theobald F (1901). A monograph of the Culicidae, or mosquitoes.

[38] Clark-Gil S, Darsie R (1983). The mosquitoes of Guatemala. Their identification, distribution and bionomics, with keys to adult females and larvae. Mosq. Syst..

[39] Darsie RF (1983). The occurrence of *Psorophora cingulata* and *Uranotaenia apicalis* in Guatemala (Diptera: Culicidae). Mosq Syst.

[40] Goindin D, Delannay C, Gelasse A, Ramdini C, Gaude T, Faucon F (2017). Levels of insecticide resistance to deltamethrin, malathion, and temephos, and associated mechanisms in *Aedes aegypti* mosquitoes from the Guadeloupe and Saint Martin islands (French West Indies). Infect Dis Poverty.

[41] Tahir D, Davoust B, Almeras L, Berenger JM, Varloud M, Parola P (2017). Anti-feeding and insecticidal efficacy of a topical administration of dinotefuran–pyriproxyfen–permethrin spot-on (Vectra® 3D) on mice against *Stegomyia albopicta* (= *Aedes albopictus*). Med Vet Entomol.

[42] Nebbak A, Koumare S, Willcox AC, Berenger JM, Raoult D, Almeras L (2017). Field application of MALDI-TOF MS on mosquito larvae identification. Parasitology.

[43] Folmer O, Black M, Hoeh W, Lutz R, Vrijenhoek R (1994). DNA primers for amplification of mitochondrial cytochrome c oxidase subunit I from diverse metazoan invertebrates. Mol Mar Biol Biotechnol.

[44] Ratnasingham S, Hebert PDN. bold: The Barcode of Life Data System (http://www.barcodinglife.org). Mol Ecol Notes. 2007;7:355–64. 10.1111/j.1471-8286.2007.01678.x PMC1890991 18784790

[45] Diarra AZ, Almeras L, Laroche M, Berenger JM, Koné AK, Bocoum Z (2017). Molecular and MALDI-TOF identification of ticks and tick-associated bacteria in Mali. PLoS Negl Trop Dis..

