# Supplementary material for: Improvement of mosquito identification by MALDI-TOF MS biotyping using protein signatures from two body parts
Source: Parasit Vectors. 2018 Nov 3;11:574. doi: 10.1186/s13071-018-3157-1 (PMC6215610; doi:10.1186/s13071-018-3157-1)
Supplement: Supplementary file 1 — Table S1. Mass peak list distinguishing mosquito species using legs as biological material, based on the Genetic Algorithm model analysis of ClinProTools. The list includes unique species-specific mass peaks. Abbreviations: Da, Daltons; m/z, mass to charge ratio. (DOCX 16 kb) [file 13071_2018_3157_MOESM1_ESM.docx]

**Additional file 1: Table S1.** Mass peak list distinguishing mosquito species using legs as biological material, based on the Genetic Algorithm model analysis of ClinProTools. The list includes unique species-specific mass peaks. *Abbreviations*: Da, Daltons; m/z, mass to charge ratio.

| Mass m/z [Da] | Start Mass | End Mass | *Ae. aegypti* | *Ae. albopictus* | *Ae. taeniorhynchus* | *Cx. atratus s.l.* | *Cx. nigripalpus* | *Cx. quinquefaciatus* | *D. magnus* | *P. cingulata* |
| --- | --- | --- | --- | --- | --- | --- | --- | --- | --- | --- |
| 2437.68 | 2433.47 | 2443.6 |  |  |  |  |  |  |  |  |
| 3263.51 | 3256.48 | 3270.33 |  |  |  |  |  |  |  |  |
| 4068.66 | 4058.13 | 4073.6 |  |  |  |  |  |  |  |  |
| 4076.39 | 4073.6 | 4083.73 |  |  |  |  |  |  |  |  |
| 4094.72 | 4086.72 | 4099.85 |  |  |  |  |  |  |  |  |
| 4107.45 | 4099.85 | 4111.81 |  |  |  |  |  |  |  |  |
| 4178.47 | 4169.46 | 4185.14 |  |  |  |  |  |  |  |  |
| 4205.69 | 4193.59 | 4215.38 |  |  |  |  |  |  |  |  |
| 4335.12 | 4327.6 | 4342.96 |  |  |  |  |  |  |  |  |
| 4880.84 | 4871.12 | 4892.64 |  |  |  |  |  |  |  |  |
| 4955.07 | 4942.39 | 4962.76 |  |  |  |  |  |  |  |  |
| 4989.63 | 4980.53 | 5000.97 |  |  |  |  |  |  |  |  |
| 5111.27 | 5095.83 | 5131.88 |  |  |  |  |  |  |  |  |
| 5179.5 | 5169.4 | 5190.23 |  |  |  |  |  |  |  |  |
| 5307.76 | 5293.65 | 5317.46 |  |  |  |  |  |  |  |  |
| 5334.38 | 5317.46 | 5344.73 |  |  |  |  |  |  |  |  |
| 5482.89 | 5473.83 | 5490.42 |  |  |  |  |  |  |  |  |
| 7398.25 | 7375.53 | 7410.88 |  |  |  |  |  |  |  |  |
| 7425.4 | 7410.88 | 7435.04 |  |  |  |  |  |  |  |  |
| 7520.79 | 7509.35 | 7526.37 |  |  |  |  |  |  |  |  |
| 7553.58 | 7541.78 | 7565.33 |  |  |  |  |  |  |  |  |
| 7629.65 | 7613.36 | 7637.84 |  |  |  |  |  |  |  |  |
| 7652.25 | 7637.84 | 7657.46 |  |  |  |  |  |  |  |  |
| 7677.19 | 7667.28 | 7709.89 |  |  |  |  |  |  |  |  |
| 8136.93 | 8114.72 | 8146.75 |  |  |  |  |  |  |  |  |
| 8153.54 | 8146.75 | 8164.47 |  |  |  |  |  |  |  |  |
| 8189.29 | 8175.45 | 8199.98 |  |  |  |  |  |  |  |  |
| 8215.03 | 8199.98 | 8222.85 |  |  |  |  |  |  |  |  |
| 8229.66 | 8222.85 | 8242.36 |  |  |  |  |  |  |  |  |
| 8357.22 | 8350.49 | 8369.29 |  |  |  |  |  |  |  |  |
| 8410.68 | 8399.25 | 8420.68 |  |  |  |  |  |  |  |  |
| 8619.71 | 8606.98 | 8626.07 |  |  |  |  |  |  |  |  |
| 8636.48 | 8626.07 | 8657.36 |  |  |  |  |  |  |  |  |
| 8670.61 | 8658.23 | 8682.6 |  |  |  |  |  |  |  |  |
| 8956.52 | 8945.91 | 8981.32 |  |  |  |  |  |  |  |  |
| 9066.21 | 9039.89 | 9092.42 |  |  |  |  |  |  |  |  |
| 9936.08 | 9923.8 | 9946.17 |  |  |  |  |  |  |  |  |
| 9988.51 | 9976.98 | 10002.22 |  |  |  |  |  |  |  |  |
| 10215.8 | 10198.67 | 10227.97 |  |  |  |  |  |  |  |  |
| 10276.67 | 10263 | 10291.45 |  |  |  |  |  |  |  |  |
| 10458.03 | 10445.74 | 10469.65 |  |  |  |  |  |  |  |  |
| 10504.2 | 10484.97 | 10529.08 |  |  |  |  |  |  |  |  |
| 10623.46 | 10614.68 | 10637.82 |  |  |  |  |  |  |  |  |
| 10650.08 | 10637.82 | 10657.13 |  |  |  |  |  |  |  |  |
| 10664.75 | 10657.13 | 10671.62 |  |  |  |  |  |  |  |  |
| 10677.8 | 10671.62 | 10691.92 |  |  |  |  |  |  |  |  |
| 10823.5 | 10807.34 | 10843.35 |  |  |  |  |  |  |  |  |
| 10962.3 | 10948.82 | 10976.24 |  |  |  |  |  |  |  |  |
| 11135.08 | 11120.8 | 11150.42 |  |  |  |  |  |  |  |  |
| 14793.17 | 14744.94 | 14817.81 |  |  |  |  |  |  |  |  |
| 14847.64 | 14817.81 | 14865.73 |  |  |  |  |  |  |  |  |
| 15102.13 | 15071.98 | 15157.19 |  |  |  |  |  |  |  |  |
| 15255.19 | 15195.26 | 15269.25 |  |  |  |  |  |  |  |  |
| 17271.96 | 17208.39 | 17316.73 |  |  |  |  |  |  |  |  |
| **Total** |  |  | **10** | **6** | **13** | **4** | **3** | **5** | **1** | **12** |
