# Supplementary material for: Improvement of mosquito identification by MALDI-TOF MS biotyping using protein signatures from two body parts
Source: Parasit Vectors. 2018 Nov 3;11:574. doi: 10.1186/s13071-018-3157-1 (PMC6215610; doi:10.1186/s13071-018-3157-1)
Supplement: Supplementary file 2 — Table S2. Mass peak list distinguishing mosquito species using thoraxes as biologic material, based on the Genetic Algorithm model analysis of ClinProTools. The list includes unique species-specific mass peaks. Abbreviations: Da, Daltons; m/z, mass to charge ratio. (DOCX 17 kb) [file 13071_2018_3157_MOESM2_ESM.docx]

**Additional file 2: Table S2.** Mass peak list distinguishing mosquito species using thoraxes as biological material, based on the Genetic Algorithm model analysis of ClinProTools. The list includes unique species-specific mass peaks. *Abbreviations*: Da, Daltons; m/z, mass to charge ratio.

| Mass m/z [Da] | Start Mass | End Mass | *Ae. aegypti* | *Ae. albopictus* | *Ae. taeniorhynchus* | *Cx. atratus s.l.* | *Cx. nigripalpus* | *Cx. quinquefaciatus* | *D. magnus* | *P. cingulata* |
| --- | --- | --- | --- | --- | --- | --- | --- | --- | --- | --- |
| 2612.92 | 2609.45 | 2616.62 |  |  |  |  |  |  |  |  |
| 2624.55 | 2617.58 | 2630.51 |  |  |  |  |  |  |  |  |
| 2671.4 | 2666.6 | 2676.26 |  |  |  |  |  |  |  |  |
| 2753.55 | 2750.3 | 2759.13 |  |  |  |  |  |  |  |  |
| 3023.44 | 3018.93 | 3029.22 |  |  |  |  |  |  |  |  |
| 3158.49 | 3152.96 | 3165.58 |  |  |  |  |  |  |  |  |
| 3178.57 | 3168.74 | 3186.13 |  |  |  |  |  |  |  |  |
| 3195.93 | 3186.13 | 3199.88 |  |  |  |  |  |  |  |  |
| 3203.18 | 3199.88 | 3207.82 |  |  |  |  |  |  |  |  |
| 3211.02 | 3207.82 | 3216.3 |  |  |  |  |  |  |  |  |
| 3252.89 | 3247.67 | 3258.34 |  |  |  |  |  |  |  |  |
| 4281.88 | 4272.06 | 4287.36 |  |  |  |  |  |  |  |  |
| 4350.95 | 4341.45 | 4356.88 |  |  |  |  |  |  |  |  |
| 4472.7 | 4468.76 | 4477.52 |  |  |  |  |  |  |  |  |
| 4594.54 | 4590.31 | 4603 |  |  |  |  |  |  |  |  |
| 4828.99 | 4822.7 | 4834.41 |  |  |  |  |  |  |  |  |
| 5095.58 | 5084.85 | 5104.9 |  |  |  |  |  |  |  |  |
| 5124.82 | 5114.26 | 5130.34 |  |  |  |  |  |  |  |  |
| 5162.22 | 5155.85 | 5171.99 |  |  |  |  |  |  |  |  |
| 5205.25 | 5198.28 | 5217.87 |  |  |  |  |  |  |  |  |
| 5230.45 | 5222.6 | 5243.59 |  |  |  |  |  |  |  |  |
| 5254.37 | 5244.27 | 5266.66 |  |  |  |  |  |  |  |  |
| 5279.69 | 5271.42 | 5289.78 |  |  |  |  |  |  |  |  |
| 5498.77 | 5484.2 | 5505.71 |  |  |  |  |  |  |  |  |
| 5511.1 | 5505.71 | 5520.31 |  |  |  |  |  |  |  |  |
| 6055.33 | 6043.39 | 6057.23 |  |  |  |  |  |  |  |  |
| 6075.87 | 6057.23 | 6079.84 |  |  |  |  |  |  |  |  |
| 6167.13 | 6155.27 | 6178.06 |  |  |  |  |  |  |  |  |
| 6235.14 | 6228.21 | 6238.56 |  |  |  |  |  |  |  |  |
| 6320.48 | 6308.27 | 6332.83 |  |  |  |  |  |  |  |  |
| 6361.15 | 6339.54 | 6381.36 |  |  |  |  |  |  |  |  |
| 6394.33 | 6382.11 | 6400.82 |  |  |  |  |  |  |  |  |
| 6407.52 | 6400.82 | 6416.56 |  |  |  |  |  |  |  |  |
| 6424.92 | 6416.56 | 6430.82 |  |  |  |  |  |  |  |  |
| 6507.14 | 6498.58 | 6519.74 |  |  |  |  |  |  |  |  |
| 6603.26 | 6600.13 | 6613.07 |  |  |  |  |  |  |  |  |
| 6908.97 | 6896.29 | 6926.65 |  |  |  |  |  |  |  |  |
| 7171.73 | 7155.33 | 7194.2 |  |  |  |  |  |  |  |  |
| 8050.06 | 8039.17 | 8057.66 |  |  |  |  |  |  |  |  |
| 8135.98 | 8120 | 8145.35 |  |  |  |  |  |  |  |  |
| 8226.51 | 8219.08 | 8253.94 |  |  |  |  |  |  |  |  |
| 8295.66 | 8285.46 | 8308.5 |  |  |  |  |  |  |  |  |
| 8323.46 | 8320.46 | 8338.42 |  |  |  |  |  |  |  |  |
| 8409.97 | 8395.84 | 8420.75 |  |  |  |  |  |  |  |  |
| 8438.06 | 8425.91 | 8453.45 |  |  |  |  |  |  |  |  |
| 8465.09 | 8453.45 | 8480.18 |  |  |  |  |  |  |  |  |
| 8527.78 | 8513 | 8541.55 |  |  |  |  |  |  |  |  |
| 8562.82 | 8548.48 | 8578.82 |  |  |  |  |  |  |  |  |
| 8700.04 | 8682.41 | 8715.62 |  |  |  |  |  |  |  |  |
| 8734.11 | 8722.62 | 8755.9 |  |  |  |  |  |  |  |  |
| 8810.93 | 8798.04 | 8832.35 |  |  |  |  |  |  |  |  |
| 8867.99 | 8842.04 | 8879.97 |  |  |  |  |  |  |  |  |
| 9410.31 | 9386.81 | 9418.61 |  |  |  |  |  |  |  |  |
| 9426.36 | 9418.61 | 9434.99 |  |  |  |  |  |  |  |  |
| 10912.12 | 10889.2 | 10948.96 |  |  |  |  |  |  |  |  |
| 12148.23 | 12111.47 | 12155.89 |  |  |  |  |  |  |  |  |
| Total |  |  | **9** | **5** | **6** | **12** | **4** | **6** | **9** | **5** |
