# Supplementary figures and images for: Improvement of mosquito identification by MALDI-TOF MS biotyping using protein signatures from two body parts
Source: Parasit Vectors. 2018 Nov 3;11:574. doi: 10.1186/s13071-018-3157-1 (PMC6215610; doi:10.1186/s13071-018-3157-1)

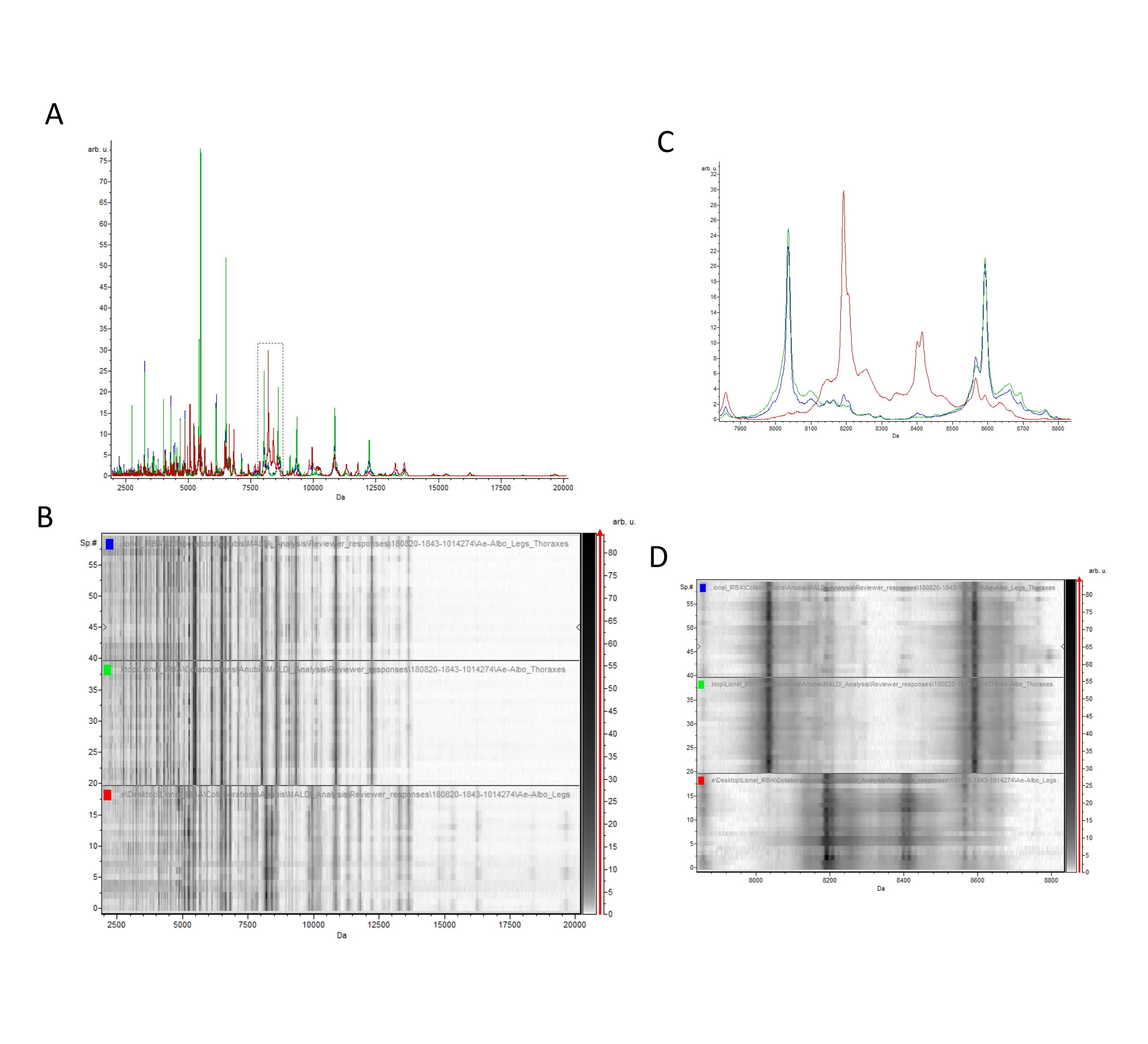

Supplement: Supplementary file 3 — Figure S1. Resulting MS spectra for legs (red), thoraxes (green) and mix of legs and thoraxes (blue) from Ae. albopictus specimens. Five specimens per condition, loaded in quadruplicate, were tested. a Overlay of the mean MS profile per condition. b Gel view of the MS profiles per condition. An enlargement of the m/z window including the more intense MS peak from Ae. albopictus legs is presented as a MS spectra overlay (c), and gel view (d). (TIF 23971 kb) [file 13071_2018_3157_MOESM3_ESM.tif]

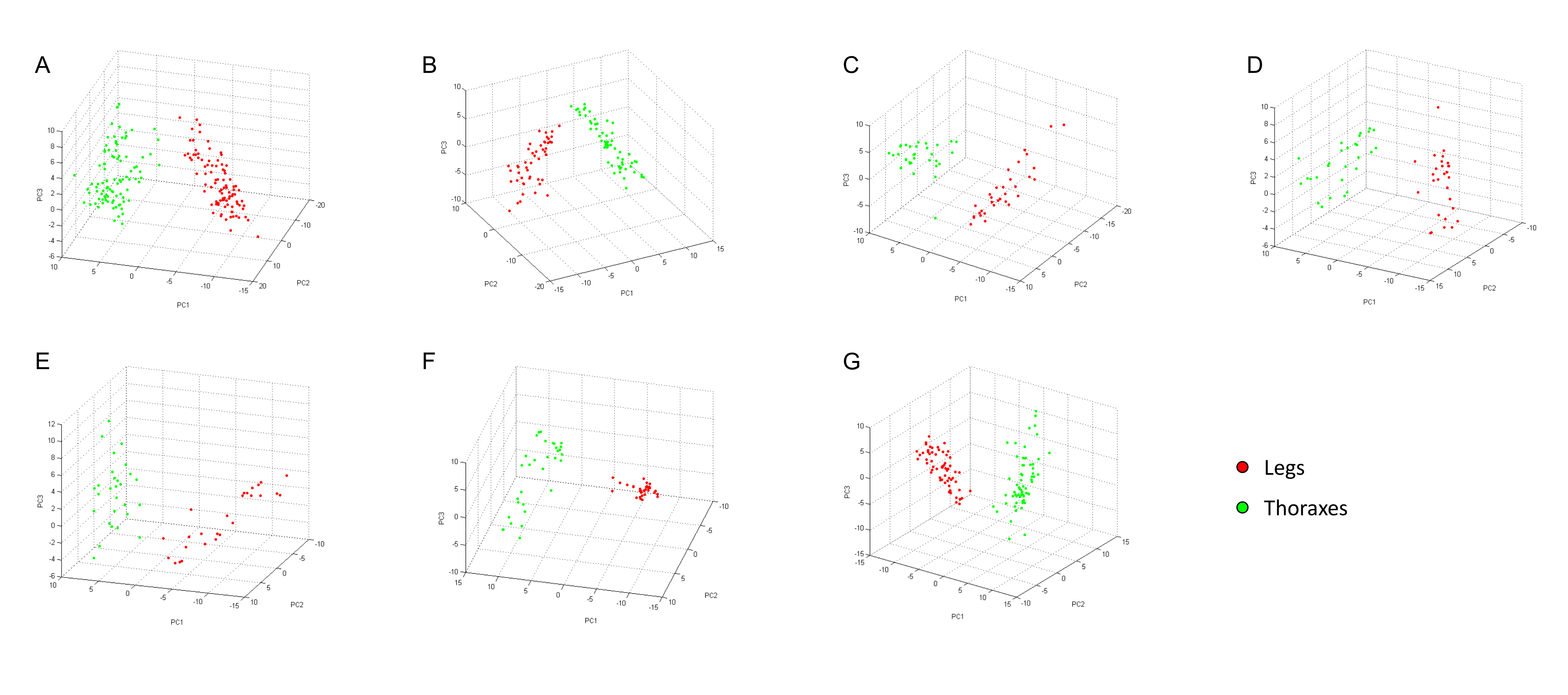

Supplement: Supplementary file 4 — Figure S2. Principal components analysis (PCA) from MS spectra for mosquito legs and thoraxes. PCA 3-dimensional (PCA1-PCA3) image from MS spectra of legs (red dots) and thoraxes (green dots) from Ae. aegypti (a), Cx. quinquefasciatus (b), Ae. taeniorynchus (c), P. cingulata (d), Cx. atratus (s.l.) (e), Cx. nigripalpus (f) and D. magnus (g). (TIF 44631 kb) [file 13071_2018_3157_MOESM4_ESM.tif]

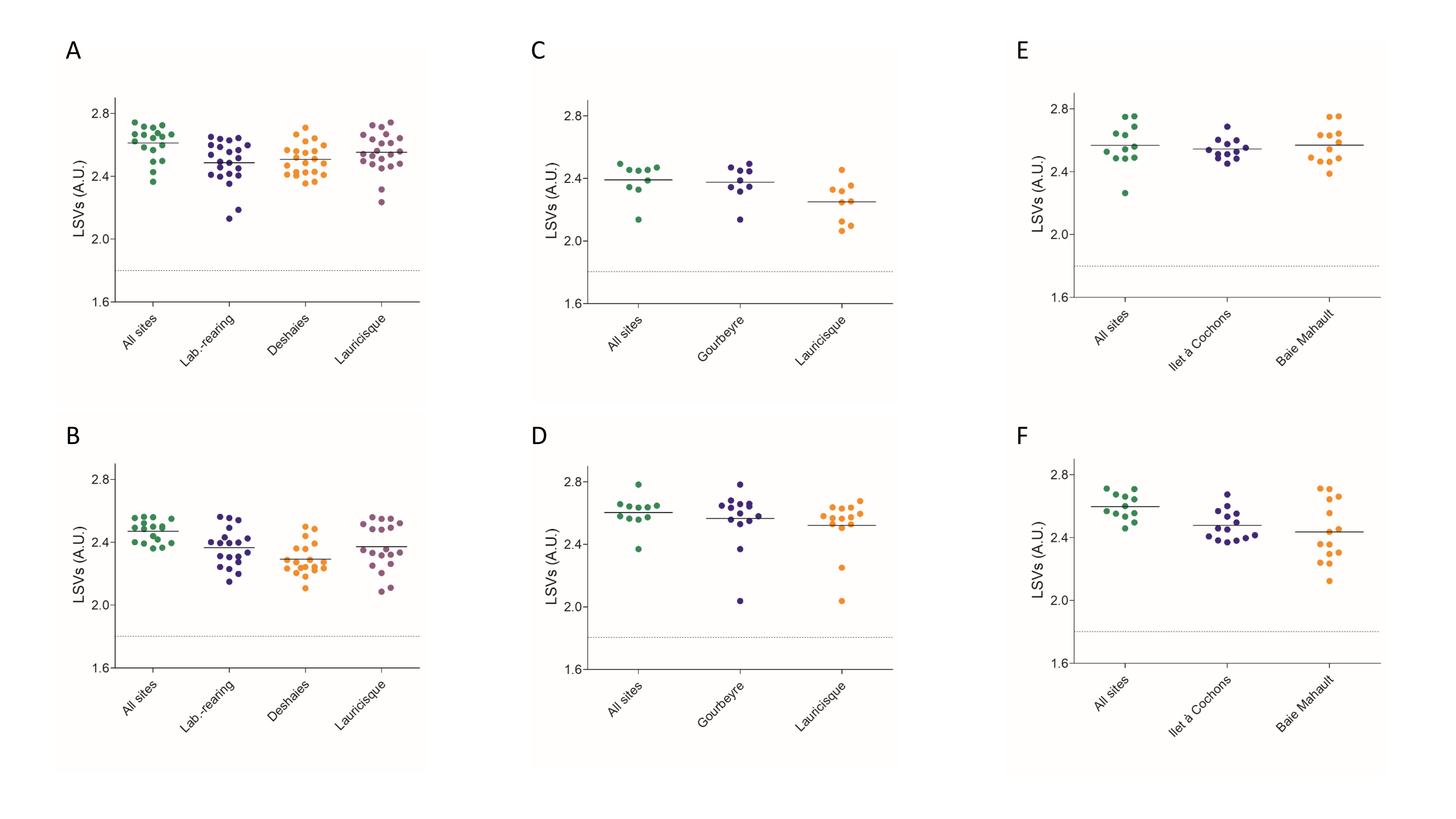

Supplement: Supplementary file 5 — Figure S3. Geographical reproducibility of the MS spectra from legs (a, c, e) and thoraxes (b, d, f) included in the DB per mosquito species. LSVs obtained for Ae. aegypti (a, b), Cx. quinquefasciatus (c, d) and D. magnus (e, f), according to origins of the MS spectra from specimens of the same species included in the DB are shown. The dashed line represents the threshold value for relevant identification (LSVs > 1.8). Abbreviation: LSV, log-score value. (TIF 35156 kb) [file 13071_2018_3157_MOESM5_ESM.tif]
