# Supplementary material for: Improvement of mosquito identification by MALDI-TOF MS biotyping using protein signatures from two body parts
Source: Parasit Vectors. 2018 Nov 3;11:574. doi: 10.1186/s13071-018-3157-1 (PMC6215610; doi:10.1186/s13071-018-3157-1)
Supplement: Supplementary file 6 — Figure S4. Experimental design for mosquito identification using two distinct compartments by MALDI-TOF MS. The advantages of the creation of a MS reference database (DB) including mosquito legs and thoraxes are indicated. (PDF 95 kb) [file 13071_2018_3157_MOESM6_ESM.pdf]

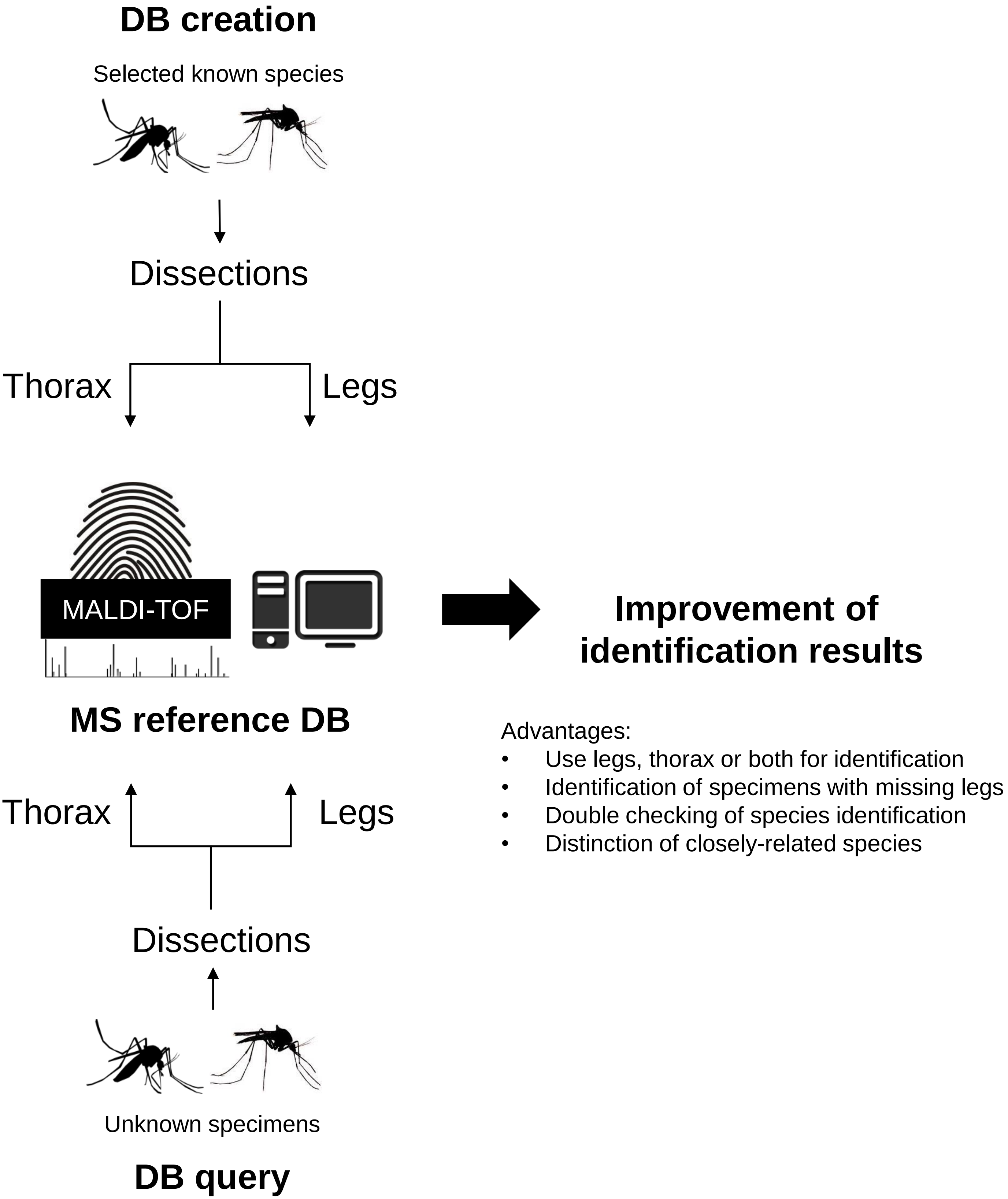
DB creation
Selected known species
Dissections
Thorax
Legs
MALDI-TOF
MS reference DB
Improvement of identification results
Advantages:
• Use legs, thorax or both for identification
• Identification of specimens with missing legs
• Double checking of species identification
• Distinction of closely-related species
Thorax
Legs
Dissections
Unknown specimens
DB query
